# Supplementary material for: Reward-predictive representations generalize across tasks in reinforcement learning
Source: PLoS Comput Biol. 2020 Oct 15;16(10):e1008317. doi: 10.1371/journal.pcbi.1008317 (PMC7591094; doi:10.1371/journal.pcbi.1008317)
Supplement: S1 Text — (PDF) [file pcbi.1008317.s001.pdf]

Reward-predictive representations generalize across tasks in reinforcement learning

Lucas Lehnert[1,3,*], Michael L. Littman[1], Michael J. Frank[2,3],

1 Computer Science Department, Brown University, Providence, RI 02912, USA
2 Department of Cognitive, Linguistic & Psychological Sciences, Brown University, Providence, RI 02912, USA
3 Carney Institute for Brain Science, Brown University, Providence, RI 02912, USA

* lucas_lehnert@brown.edu

Supporting information

S.1 State Abstractions

For finite state and action MDPs, the transition function can be presented as a set of left-stochastic transition matrices $\{\boldsymbol{M}_a\}_{a \in \mathcal{A}}$ where

$$\forall a \in \mathcal{A},\ \forall i, j \in \mathcal{S},\ \boldsymbol{M}_a(i,j) = p(i,a,j), \tag{1}$$

and a set of expected reward vectors $\{\boldsymbol{r}_a\}_{a \in \mathcal{A}}$ where

$$\forall a \in \mathcal{A},\ \forall i \in \mathcal{S},\ \boldsymbol{r}_a(i) = \mathbb{E}_{s'}[r(s,a,s')]. \tag{2}$$

A state abstraction is an MDP homomorphism [1] mapping an MDP $M = \langle \mathcal{S}, \mathcal{A}, p, r, \gamma \rangle$ into an abstract MDP $M_\phi = \langle \mathcal{S}_\phi, \mathcal{A}, m, w, \gamma \rangle$. Using a weighting function ω, the abstract MDP "aggregates" the transition and reward function across states $s \in \mathcal{S}$ that map to the same abstract state $s_\phi \in \mathcal{S}_\phi$. Specifically, the abstract reward function w is constructed as

$$w(s_\phi, a) = \sum_{s:\phi(s)=s_\phi} \omega(s) \boldsymbol{r}_a(s). \tag{3}$$

The transition function between latent states m is constructed as

$$m(s_\phi, a, s'_\phi) = \sum_{s:\phi(s)=s_\phi} \sum_{s':\phi(s')=s'_\phi} \omega(s) p(s,a,s'). \tag{4}$$

The weighting function is assumed to be non-negative and the sum across a state partition evaluates to one: $\sum_{s:\phi(s)=s_\phi} \omega(s) = 1$. The presented simulations assume a uniform weighting function which averages across state partitions. Similar to Eq (1) and Eq (2), the abstract transition matrices $\{\boldsymbol{M}_a\}_{a\in\mathcal{A}}$ and abstract reward vectors $\{\boldsymbol{w}_a\}_{a\in\mathcal{A}}$ can be computed and

$$\forall a \in \mathcal{A},\ \forall i,j \in \mathcal{S}_\phi,\ \boldsymbol{M}_a(i,j) = m(i,a,j) \text{ and } \boldsymbol{w}_a(i) = w(i,a). \tag{5}$$

Note that function m defines transition probabilities between latent states in the same way the transition function p defines transition probabilities between states [2]. Consequently, the latent model described by the matrices and vectors $\{\boldsymbol{M}_a, \boldsymbol{w}_a\}_{a\in\mathcal{A}}$ can be used as a normal MDP, with the only difference that this latent MDP is defined on latent states. A policy that is optimal with respect to this compressed or latent MDP $\{\boldsymbol{M}_a, \boldsymbol{w}_a\}_{a\in\mathcal{A}}$ can be computed by performing value iteration [3, Chapter 4.4] on the matrices and vectors $\{\boldsymbol{M}_a, \boldsymbol{w}_a\}_{a\in\mathcal{A}}$. Such a policy is then used as the optimal policy for an agent that generalizes across states according to the state abstraction ϕ.

References

1. Ravindran B, Barto AG. Approximate Homomorphisms: A framework for non-exact minimization in Markov Decision Processes. 2004;.

2. Li L, Walsh TJ, Littman ML. Towards a Unified Theory of State Abstraction for MDPs. In: ISAIM; 2006.

3. Sutton RS, Barto AG. Reinforcement learning: An introduction. MIT press; 2018.
