# Supplementary material for: Reward-predictive representations generalize across tasks in reinforcement learning
Source: PLoS Comput Biol. 2020 Oct 15;16(10):e1008317. doi: 10.1371/journal.pcbi.1008317 (PMC7591094; doi:10.1371/journal.pcbi.1008317)
Supplement: S2 Text — (PDF) [file pcbi.1008317.s002.pdf]

Reward-predictive representations generalize across tasks in reinforcement learning

Lucas Lehnert[1,3,*], Michael L. Littman[1], Michael J. Frank[2,3],

1 Computer Science Department, Brown University, Providence, RI 02912, USA
2 Department of Cognitive, Linguistic & Psychological Sciences, Brown University, Providence, RI 02912, USA
3 Carney Institute for Brain Science, Brown University, Providence, RI 02912, USA

* lucas_lehnert@brown.edu

Supporting information

S.2 Successor Features Identify Reward-Predictive State Abstractions

Linear Successor Feature Models (LSFMs) can be used to identify reward-predictive state abstractions [1]. Critically, LSFMs construct a state abstraction ϕ so as to most accurately predict the SFs at every state s and action a by requiring that

$$\boldsymbol{F}_a \phi(s) \approx \boldsymbol{\psi}^{\pi}(s,a) \tag{1}$$

holds for a set of real-valued square matrices $\{\boldsymbol{F}_a\}_{a \in \mathcal{A}}$. Intuitively, Eq. (1) asserts that a state feature vector $\phi(s)$ has to be constructed such that only a linear transform can be used to predict the SFs $\boldsymbol{\psi}^{\pi}(s,a)$. By [1, Lemma 1], requiring a state abstraction to predict the SFs $\boldsymbol{\psi}^{\pi}$ is equivalent to requiring a state abstraction to predict the expected next state feature vector (the vector $\mathbb{E}[\phi(s')|s,a]$ that is reached by selecting action a at state s). In fact, this model, called Linear Action Model (LAM), can also be used to learn reward-predictive state abstraction [1]. While both models can be used to learn reward-predictive state abstractions, LSFMs and LAMs are not equivalents of one another and we have found it easier to use LSFMs in practice. If such a state abstraction also associates each feature vector $\phi(s_t)$ with one-step reward outcomes, then this state abstraction is reward predictive. Similar to Eq. (1), this property can be

enforced by requiring for some set of real valued vectors $\{\boldsymbol{w}_a\}_{a \in \mathcal{A}}$ that

$$\boldsymbol{w}_a^\top \phi(s) \approx r(s,a), \tag{2}$$

where $r(s,a)$ is the one-step expected reward that can be obtained by selecting action a at state s. To obtain a state abstraction that satisfies Eqs. (1) and (2), the differences between the left-hand and right-hand sides of Eqs. (1) and (2):

$$l_{\text{LSFM}}(\phi) = \sum_{s,a} c \underbrace{||\boldsymbol{F}_a \phi(s) - \boldsymbol{\psi}^\pi(s,a)||_2^2}_{=\varepsilon_\psi} + \underbrace{|\boldsymbol{w}_a^\top \phi(s) - r(s,a)|}_{=\varepsilon_r}, \tag{3}$$

where c is a real-valued constant (we denote the Euclidean L2 norm with $||\cdot||_2$). For finite MDPs, the summation in Eq. (3) is computed over all possible states and actions. (Please refer to supplemental material S2 for a detailed description.) Finding a state abstraction ϕ that minimizes the loss objective l_{LSFM} is equivalent to learning reward-predictive state abstractions [1, Theorem 2 and Lemma 1], because

$$\left|\phi^\top(s)\boldsymbol{M}_{a_1}\cdots\boldsymbol{M}_{a_{t-1}}\boldsymbol{w}_{a_t} - \mathbb{E}[r_t|s,a_1,...,a_t]\right| \leq \varepsilon_r + C_{\gamma,t}\varepsilon_\psi \tag{4}$$

where $C_{\gamma,t}$ is a constant depending on the discount factor γ and sequence length T. In this article all abstract states $\phi(s)$ are encoded as one-hot bit vectors, a vector of zeros with only one entry set to one. The product $\phi^\top(s)\boldsymbol{M}_{a_1}\cdots\boldsymbol{M}_{a_{t-1}}$ evaluates to a row probability vector specifying with which probability an abstract state is reached starting at the abstract state $\phi(s)$ and following the action sequence $a_1,...,a_{t-1}$. By computing the dot-product between this vector and the reward vector $\boldsymbol{w}_{a_t}$, the prediction of the expected reward is calculated after following the actions sequence $a_1,...,a_t$ starting at state s with $\phi^\top(s)\boldsymbol{M}_{a_1}\cdots\boldsymbol{M}_{a_{t-1}}\boldsymbol{w}_{a_t}$. The loss function $l_{\text{predictive}}$ is then computed with

$$l_{\text{predictive}}(\phi) = \varepsilon_r + \varepsilon_\psi. \tag{5}$$

If $l_{\text{predictive}}(\phi) = 0$, the ϕ is a reward-predictive state abstraction. A more detailed description of these bounds and LSFMs was previously presented by [1].

Transferring SFs Across Tasks

To establish a baseline and investigate to what extend re-using previously learned Q-values or SFs accelerates learning, supporting S1 Fig re-produces the navigation experiment presented in prior work [3] and demonstrates the benefit of transferring SFs between different tasks as in prior work [4–7]. In this experiment, the Q-learning [8] and SF-learning [4] algorithms are tested on a sequence of four grid-world navigation tasks (supporting S1A Fig). SF-learning is an algorithm that learns a SF vector $\boldsymbol{\psi}^{\pi}(s, a)$ for each state s and action a. Similar to Q-learning, SF-learning iteratively improves its current behaviour policy until an optimal policy is found [4]. In supporting text S3 we present all implementation details on how these algorithms are implemented and evaluated. Each agent was allowed to attempt each navigation task for 200 episodes. After 200 episodes, each agent is signalled that the grid-world map has changed. While interacting with a task, each algorithm updates its internal values and attempts to find an optimal policy that reaches the goal location as fast as possible.

Supporting S1B Fig plots the episode length of the SF-learning algorithm as a function of the number of episodes. The gray lines indicate when the interaction in one of the four tasks begins. This plot shows that at the end of learning in each task, a close to optimal policy is found because the agent can repeatedly navigate from start to goal location within about ten time steps. However, at the beginning of learning in each map (episode 0, 200, 400, and 600), the episode length spikes up. The first spike at episode zero occurs because the SF-learning algorithm is initialized and has to learn an optimal policy from scratch. At episodes 200, 400, and 600, the SF-learning algorithm is reset but is allowed to preserve its previously learned SFs and reward weight vector. Because the change in goal location also leads to a change in optimal policy, the episode length spikes up when the goal location is changed, but the SF-learning algorithm can subsequently recover an optimal policy for that new task [9].

Supporting S1C Fig plots the average episode length for each task and tested algorithm. In this plot, each point is averaged across the 200 episodes an agent spent in each task. Because the task is to navigate across a grid world, a shorter episode or trial length is indicative of a policy that generates more reward per time step. For both Q-learning and SF-learning the average episode length does not vary significantly across

the different tasks. This behaviour is expected, because both algorithms are reset when the grid map is changed and an optimal policy is learned from scratch. Furthermore, these simulations indicate that SF-learning converges to an optimal policy faster than Q-learning in this particular experiment, because the average episode length is significantly lower for SF-learning than for Q-learning. The orange curve in supporting S1C Fig plots the episode length when Q-learning re-uses previously learned Q-values. While re-using previously learned Q-values reduces the average episode length on tasks two and three, re-using Q-values does not result in a significant performance improvement and this algorithm is outperformed by the SF-learning algorithm. The red curve in supporting S1C Fig plots the episode length when the SF-learning algorithm can re-use previously learned SFs and a previously learned reward vector. In this case, the average episode length is significantly lower on tasks two through four when the SF-learning algorithm is allowed to transfer previously learned SFs. While SFs have to be re-learned [4, 9], re-using previously learned SFs leads to faster convergence. This experiment aligns with previous findings demonstrating that re-using SFs can speed up learning [3, 4, 6, 7, 9–13].

References

1. Lehnert L, Littman ML. Successor Features Combine Elements of Model-Free and Model-based Reinforcement Learning. arXiv preprint arXiv:190111437v2. 2019;.

2. Dayan P. Improving generalization for temporal difference learning: The successor representation. Neural Computation. 1993;5(4):613–624.

3. Lehnert L, Tellex S, Littman ML. Advantages and Limitations of using Successor Features for Transfer in Reinforcement Learning. arXiv preprint arXiv:170800102. 2017;.

4. Barreto A, Dabney W, Munos R, Hunt JJ, Schaul T, van Hasselt HP, et al. Successor features for transfer in reinforcement learning. In: Advances in neural information processing systems; 2017. p. 4055–4065.

5. Barreto A, Munos R, Schaul T, Silver D. Successor Features for Transfer in Reinforcement Learning. CoRR. 2016;abs/1606.05312.

6. Momennejad I, Russek EM, Cheong JH, Botvinick MM, Daw N, Gershman SJ. The successor representation in human reinforcement learning. Nature Human Behaviour. 2017;1(9):680.

7. Russek EM, Momennejad I, Botvinick MM, Gershman SJ, Daw ND. Predictive representations can link model-based reinforcement learning to model-free mechanisms. PLoS computational biology. 2017;13(9):e1005768.

8. Watkins CJCH, Dayan P. Q-learning. Machine Learning. 1992;8(3):279–292.

9. Barreto A, Borsa D, Quan J, Schaul T, Silver D, Hessel M, et al. Transfer in Deep Reinforcement Learning Using Successor Features and Generalised Policy Improvement. In: Proceedings of the 35th International Conference on Machine Learning. vol. 80 of Proceedings of Machine Learning Research. PMLR; 2018. p. 501–510.

10. Stachenfeld KL, Botvinick MM, Gershman SJ. The hippocampus as a predictive map. Nature Neuroscience. 2017;20:1643 EP –.

11. Zhang J, Springenberg JT, Boedecker J, Burgard W. Deep reinforcement learning with successor features for navigation across similar environments. In: 2017 IEEE/RSJ International Conference on Intelligent Robots and Systems (IROS). IEEE; 2017. p. 2371–2378.

12. Kulkarni TD, Saeedi A, Gautam S, Gershman SJ. Deep successor reinforcement learning. arXiv preprint arXiv:160602396. 2016;.

13. Madarasz TJ, Behrens T. Better Transfer Learning Through Inferred Successor Maps. 2019;.
