# Supplementary material for: Reward-predictive representations generalize across tasks in reinforcement learning
Source: PLoS Comput Biol. 2020 Oct 15;16(10):e1008317. doi: 10.1371/journal.pcbi.1008317 (PMC7591094; doi:10.1371/journal.pcbi.1008317)
Supplement: S3 Text — (PDF) [file pcbi.1008317.s003.pdf]

Reward-predictive representations generalize across tasks in reinforcement learning

Lucas Lehnert[1,3,*], Michael L. Littman[1], Michael J. Frank[2,3],

1 Computer Science Department, Brown University, Providence, RI 02912, USA
2 Department of Cognitive, Linguistic & Psychological Sciences, Brown University, Providence, RI 02912, USA
3 Carney Institute for Brain Science, Brown University, Providence, RI 02912, USA

* lucas_lehnert@brown.edu

Supporting information

S.3 Hyper-Parameter Selection and Implementation of Learning Experiments

In this section we present implementation details necessary to reproduce the learning experiments presented in Fig 7, Fig 8, and supporting S1 Fig. Because the Q-learning algorithm and SF-learning algorithm use a learning rate parameter, the resulting episode lengths are sensitive to hyper-parameters and learning rates. To account for this dependency, each algorithm was tested on a range of different hyper-parameter combinations and the hyper-parameter setting for each algorithm that leads to the best performance was used for the presented experiments.

Optimistic initialization and exploration

To ensure that each algorithm learns an optimal policy using only interaction data, each algorithm was initialized optimistically to ensure efficient exploration [1]. In optimistic initialization, all Q-values [2, 3] are initialized to a specified maximum value $Q_{\max}$, which upper-bounds the actual Q-values of the task. A Q-learning agent then learns to incrementally decrease its value predictions until prediction errors are low. By combining this initialization scheme with an exploration policy that is greedy with respect to the current Q-value predictions, the Q-learning agent will repeat actions where Q-value predictions overestimate the actual return. All tested tasks are

navigation tasks where an agent always receives a reward of one once the goal location is reached and a reward of zero otherwise. Because the reward of one is incurred only once, all Q-values lie in the interval $[0, 1]$ and $Q_{\max} = 1$.

Supporting S2 Fig demonstrates that optimistic initialization combined with a greedy action selection strategy significantly outperforms initializing Q-values or SFs to zero and using an ε-greedy exploration policy as prior work did [4–6]. This result confirms that both Q-learning and SF-learning benefit from the optimistic initialization strategy used in the presented simulation experiments.

Q-learning

The Q-learning algorithm [3] was tested for three different learning rate settings: 0.1, 0.5, and 0.9. In all experiments, using a learning rate of 0.9 leads to the fastest learning and overall best performance, even when the algorithm was combined with a state abstraction. Only in supporting S2A Fig, the Q-learning baseline (without no transfer) used a learning rate of 0.7. When Q-learning was used in combination with a state abstraction, each transition (s, a, r, s') was mapped to a transition $(\phi(s), a, r', \phi(s'))$ between latent states.

SF-learning

Algorithm 1 outlines an adaptation of the SF-learning algorithm [5] that incorporates optimistic initialization. Our implementation of SF-learning maintains a matrix $\boldsymbol{G}$ of size $SA \times SA$, where S is the number of states and A the number of actions. Each row of this matrix $\boldsymbol{G}$ approximates the SF

$$\boldsymbol{G}(s, a) \approx \mathbb{E}\left[\boldsymbol{e}_{s_1,a_1} + \gamma \boldsymbol{e}_{s_2,a_2} + \gamma^2 \boldsymbol{e}_{s_3,a_3} + \cdots \middle| s_1 = s, a_1 = a, \pi\right], \tag{1}$$

where the expectation in Eq (1) is defined over all infinite length trajectories that start with state s and action a and then follow the policy π. Each vector $\boldsymbol{e}_{s_i,a_i}$ is a one-hot bit vector associated with the state-action pair (s_i, a_i). This vector has a dimension of SA and the entry corresponding to the state action pair (s_i, a_i) is set to one with all other entries being zero. The definition presented in Eq (1) is equivalent to the definition presented by [5], with the distinction that one-hot bit vector features are used.

Consider a vector $\boldsymbol{v}$ of length SA storing one-step reward predictions in each entry:

$$\boldsymbol{v}(s,a) = r(s,a) \tag{2}$$

Algorithm 1 incrementally learns a matrix $\boldsymbol{G}$ and vector $\boldsymbol{v}$ such that Eq (1) and Eq (2) are satisfied. The iterates in lines 8 and 9 of Algorithm 1 are obtained by substituting $\boldsymbol{\phi}_t = \boldsymbol{e}_{s_t,a_t}$ and $\psi^\pi(s,a) = \boldsymbol{e}_{s,a}^\top \boldsymbol{G} = \boldsymbol{G}(s,a)$ into [6, Algorithm 1].

If the matrix $\boldsymbol{G}$ is constructed such that Eq (1) exactly holds, then [5]

$$Q^\pi(s,a) = (\boldsymbol{G}(s,a))^\top \boldsymbol{v}. \tag{3}$$

The SF-learning algorithm learns an optimal policy by constructing Q-value prediction using Eq (3). Optimistic initialization can be integrated into SF-learning by initializing the matrix $\boldsymbol{G}$ and vector $\boldsymbol{v}$ such that $Q(s,a) = Q_{\max}$ at the beginning of training. This can be accomplished by initializing $\boldsymbol{G} = \boldsymbol{I}$ and every entry $\boldsymbol{v}_0(s,a) = Q_{\max}$ (line 2 in Algorithm 1). Hence, we have for the Q-values at initialization

$$Q(s,a) = (\boldsymbol{G}(s,a))^\top \boldsymbol{v} = \boldsymbol{e}_{s,a}^\top \mathbf{1} Q_{\max} = Q_{\max}, \tag{4}$$

where $\mathbf{1}$ is a vector with each entry being set to one. By combining this initialization scheme and selecting actions greedily with the predicted Q-values, we found that the SF-learning algorithm converges faster to the Q-learning algorithm. Table A lists the tested learning rates and the best performing learning rate setting for each simulation.

Table A. Learning rates used for SF-learning.

	Tested α_{SF}	Tested α_r	Best α_{SF}	Best α_r
SF-learning with SF transfer, orange curve in Fig. 7B	0.1, 0.5, 0.9	0.1, 0.5, 0.9	0.5	0.9
SF-learning baseline, blue curve in Fig. 7B	0.1, 0.5, 0.9	0.1, 0.5, 0.9	0.5	0.9
Reward predictive with SF, green curve in Fig. 7B	0.1, 0.5, 0.9	0.1, 0.5, 0.9	0.5	0.9
SF-learning in S1 Fig and S2A Fig	0.1, 0.3, 0.5, 0.7, 0.9	0.1, 0.3, 0.5, 0.7, 0.9	0.3	0.9
SF-learning with SF transfer in S2A Fig	0.1, 0.3, 0.5, 0.7, 0.9	0.1, 0.3, 0.5, 0.7, 0.9	0.3	0.9
SF-learning with SF and reward transfer in S1 Fig and S2A Fig	0.1, 0.3, 0.5, 0.7, 0.9	0.1, 0.3, 0.5, 0.7, 0.9	0.3	0.9
SF-learning in S2B Fig	0.1, 0.3, 0.5, 0.7, 0.9	0.1, 0.3, 0.5, 0.7, 0.9	0.1	0.5
SF-learning with SF transfer in S2B Fig	0.1, 0.3, 0.5, 0.7, 0.9	0.1, 0.3, 0.5, 0.7, 0.9	0.3	0.3
SF-learning with SF and reward transfer in S2B Fig	0.1, 0.3, 0.5, 0.7, 0.9	0.1, 0.3, 0.5, 0.7, 0.9	0.1	0.7

Algorithm 1 SF-learning with optimistic initialization, adopted from [5]

```
Input: Learning rates α_SF, α_r ∈ (0,1], the maximum Q-value Q_max.
Initialize G ← I and v ← 1 ∗ Q_max where 1 is a vector of ones.
loop
    Receive current state s from MDP.
    Select action a = arg max_a' Q(s, a') with Eq (3).
    Observe transition (s, a, r, s').
    a* ← arg max_a' Q(s', a') with Eq (3).
    G ← G + α_SF(e_sa + γG(s', a*) − G(s, a))e_{s,a}^⊤
    v ← v + α_r(v(s, a) − r)e_{s,a}
end loop
```

Learning of Reward-Maximizing and Reward-Predictive State Abstractions

The learning simulations (presented in sub-section Learning to transfer multiple state abstractions) learn reward-maximizing state abstractions by clustering Q-values. These Q-values are computed by performing value iteration on the constructed transition and reward tables. By clustering Q-values a state abstraction ϕ is obtained with a low $l_{\text{maximizing}}(\phi)$ value[1] [7].

A reward-predictive state abstraction is computed by computing a LSFM with low prediction errors. Such an LSFM is obtained by using a rescaled version of the loss objective $\mathcal{L}_{\text{LSFM-mat}}$ presented by [8]:

$$\mathcal{L}_{\text{LSFM-mat-scaled}} = \sum_{a \in \mathcal{A}} \alpha_w ||\boldsymbol{\Phi} \boldsymbol{w}_a - \widehat{\boldsymbol{r}}_a||_2^2 + \alpha_F ||\boldsymbol{\Phi} + \gamma \widehat{\boldsymbol{P}}_a \boldsymbol{\Phi} \overline{\boldsymbol{F}} - \boldsymbol{\Phi} \boldsymbol{F}_a||_2^2, \quad (5)$$

where $\{\widehat{\boldsymbol{P}}_a, \widehat{\boldsymbol{r}}_a\}_{a \in \mathcal{A}}$ are the transition and reward tables the non-parametric Bayesian model computes from the observed interaction data. Each row of the matrix $\boldsymbol{\Phi}$ stores a latent feature vector that is associated with a particular state. The loss objective $\mathcal{L}_{\text{LSFM-mat-scaled}}$ is optimized using the Adam gradient optimizer [9] with a learning rate of 0.01 and $\alpha_F = 0.01$. Otherwise Tensorflow's [10] default parameters were used and the optimization process was initialized as described by [8]. After 1000 gradient update steps, the row-space of the real-valued matrix $\boldsymbol{\Phi}$ was clustered using agglomerative clustering to construct a partitioning of the state space and a state abstraction.

The resulting state representation will then have a low one-step-reward-prediction error ε_r and SF error ε_ψ and a low loss value $l_{\text{predictive}}(\phi) = \varepsilon_r + \varepsilon_\psi$. During learning in

[1] If each state partition has Q-values that differ by at most ε from another, then $l_{\text{maximizing}}(\phi) \leq 2\varepsilon/(1-\gamma)^2$.

a particular task, these loss values are computed on an incomplete transition table to compute the posterior over state abstraction needed for the mixture policy described in Eq (12) in the main article.

Hyper-Parameters Influence Belief Space Size

Supporting S3 Fig plots the average episode length and belief space size for different α and β settings for each model for the learning simulation presented in Fig 7. Supporting S3A Fig and S3C Fig indicate that a decrease in α corresponds in most cases to a smaller belief space size. As the parameter α is decreased, the CRP prior will emphasize re-use of previously learned state representations, leading to a smaller belief space size after observing all five tasks. Nevertheless, the β parameter also influence the belief space size. For high β parameters, the scores $l_{\text{predictive}}$ or $l_{\text{maximizing}}$ influence the posterior more strongly and if the loss values are low enough another state abstraction is added into the belief set despite the CRP prior. Consequently, a higher β parameter corresponds to a larger belief set size as plotted in the right panels of supporting S3 Fig. S3B Fig and S3D Fig show that a small belief space (where $\beta = 1$) results in a high average episode length and thus poor performance on the tested task sequence. If the α parameter is increased for $\beta = 1$, then the average episode length decreases because the model tends to use multiple state abstractions in its belief set instead of enforcing the re-use of the same state abstraction. If the same state abstraction is re-used on all tasks, then the used state abstraction may remove information from the state that is needed to find an optimal policy, resulting in poor performance. For both models, the average episode length is lowest when the learned belief space has a small but not too small size. This suggests that learning and re-using a small set of state abstractions results in a shorter average episode length and better performance. If the belief space is too large, then the average episode length also increases because state abstractions are not re-used to accelerate learning in subsequent tasks and the model does not transfer previously learned structures. Note that for a setting of $\beta = 100$ only the reward-predictive model constructs a belief set of size two for $\alpha \leq 0.01$ for all ten repeats. Because the task sequence was constructed by adding irrelevant state variables in one of two ways, this result suggests that the reward-predictive model detects these two generalization patterns.

Table B outlines the tested and best mixture model parameters for the simulations presented in Fig 7.

Table B. Hyper-parameters tested for mixture model.

	Tested α	Tested β	Best α	Best β
Reward maximizing, orange curve in Fig 7A	$10^{-5}, 10^{-4}, 10^{-3}, 10^{-2}, 10^{-1}$	1, 10^2, 10^4	10^{-3}	10^2
Reward predictive, green curve in Fig 7A	$10^{-5}, 10^{-4}, 10^{-3}, 10^{-2}, 10^{-1}$	1, 10^2, 10^4	10^{-5}	10^2
Reward predictive with SF-learning, green curve in 7B	10^{-9}, 10^{-5}, 10^{-1}	1, 10^2, 10^4	10^{-9}	10^2

Hyper-Parameter for Guitar Task Experiment

In the simulation presented in Fig 8, each learning algorithm interacted with with each task for 50 episodes (trials). We make the assumption that same note cannot be repeated in the scale. This assumption ensures for simplicity that the fret board can be used as a Markovian state. For each algorithm, learning on the first task was initialized by setting both the $\boldsymbol{v}$ vector and the $\boldsymbol{G}$ matrix (defined in Eq (1) and (2)) were set to zero. On task 2, values were also reset to zero for the baseline algorithm. The reward-predictive model (green curve in Fig 8) only re-used a previously learned reward-predictive state abstraction and all latent SFs and reward weight vectors were also reset to zero at the beginning of training in task 2. The SF transfer algorithm (orange curve in Fig 8) re-used the previously learned SF matrix $\boldsymbol{G}$ and the reward weight vector $\boldsymbol{w}$ was reset to zero, similar to [6].

The SF transfer algorithm and the reward-predictive model used a learning rate of $\alpha_{\text{SF}} = 0.9$ and $\alpha_r = 0.9$. The SF-learning baseline algorithm used a learning rate of $\alpha_{\text{SF}} = 0.1$ and $\alpha_r = 0.9$. A total of nine learning rate combinations were tested by setting each learning rate to either 0.1, 0.5, or 0.9.

References

1. Kaelbling LP, Littman ML, Moore AW. Reinforcement learning: A survey. Journal of artificial intelligence research. 1996;4:237–285.

2. Sutton RS, Barto AG. Reinforcement learning: An introduction. MIT press; 2018.

3. Watkins CJCH, Dayan P. Q-learning. Machine Learning. 1992;8(3):279–292.

4. Barreto A, Dabney W, Munos R, Hunt JJ, Schaul T, van Hasselt HP, et al. Successor features for transfer in reinforcement learning. In: Advances in neural information processing systems; 2017. p. 4055–4065.

5. Barreto A, Munos R, Schaul T, Silver D. Successor Features for Transfer in Reinforcement Learning. CoRR. 2016;abs/1606.05312.

6. Lehnert L, Tellex S, Littman ML. Advantages and Limitations of using Successor Features for Transfer in Reinforcement Learning. arXiv preprint arXiv:170800102. 2017;.

7. Abel D, Hershkowitz D, Littman M. Near Optimal Behavior via Approximate State Abstraction. In: Proceedings of The 33rd International Conference on Machine Learning; 2016. p. 2915–2923.

8. Lehnert L, Littman ML. Successor Features Combine Elements of Model-Free and Model-based Reinforcement Learning. arXiv preprint arXiv:190111437v2. 2019;.

9. Kingma DP, Ba J. Adam: A Method for Stochastic Optimization. CoRR. 2014;abs/1412.6980.

10. Abadi M, Agarwal A, Barham P, Brevdo E, Chen Z, Citro C, et al.. TensorFlow: Large-Scale Machine Learning on Heterogeneous Systems; 2015. Available from: https://www.tensorflow.org/.
