# Supplementary figures and images for: Reward-predictive representations generalize across tasks in reinforcement learning
Source: PLoS Comput Biol. 2020 Oct 15;16(10):e1008317. doi: 10.1371/journal.pcbi.1008317 (PMC7591094; doi:10.1371/journal.pcbi.1008317)

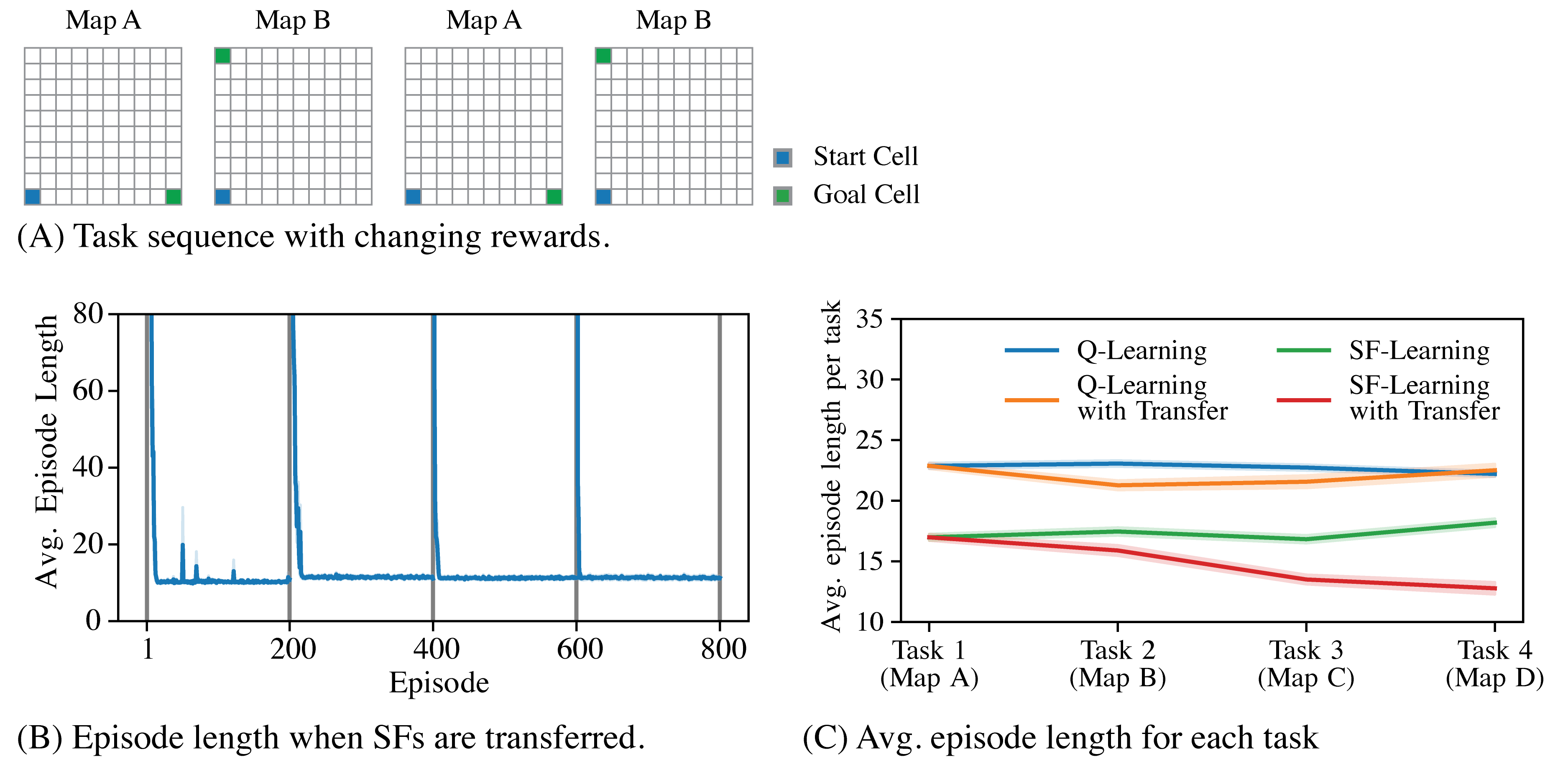

Supplement: S1 Fig — (A) In this experiment each algorithm was simulated on a sequence of grid-world maps. For each grid map, the agent starts at the blue grid cell and navigates to the green goal cell to collect a reward. The transitions are the same as described in Fig 6, but these tasks do not contain any barriers. (B) Plot of the average episode length as a function of the episode for the SF-learning agent. The gray lines indicate the start or end of learning in one of the four tasks. After a certain number of episodes, the SF-learning algorithm can find an optimal policy that navigates across the map in about ten time steps. (C) Plot of the average episode length for each task and each tested algorithm. Each simulation was repeated 20 times and averages across repeats are plotted. Standard errors indicated by the shaded areas. (TIF) [file pcbi.1008317.s004.tif]

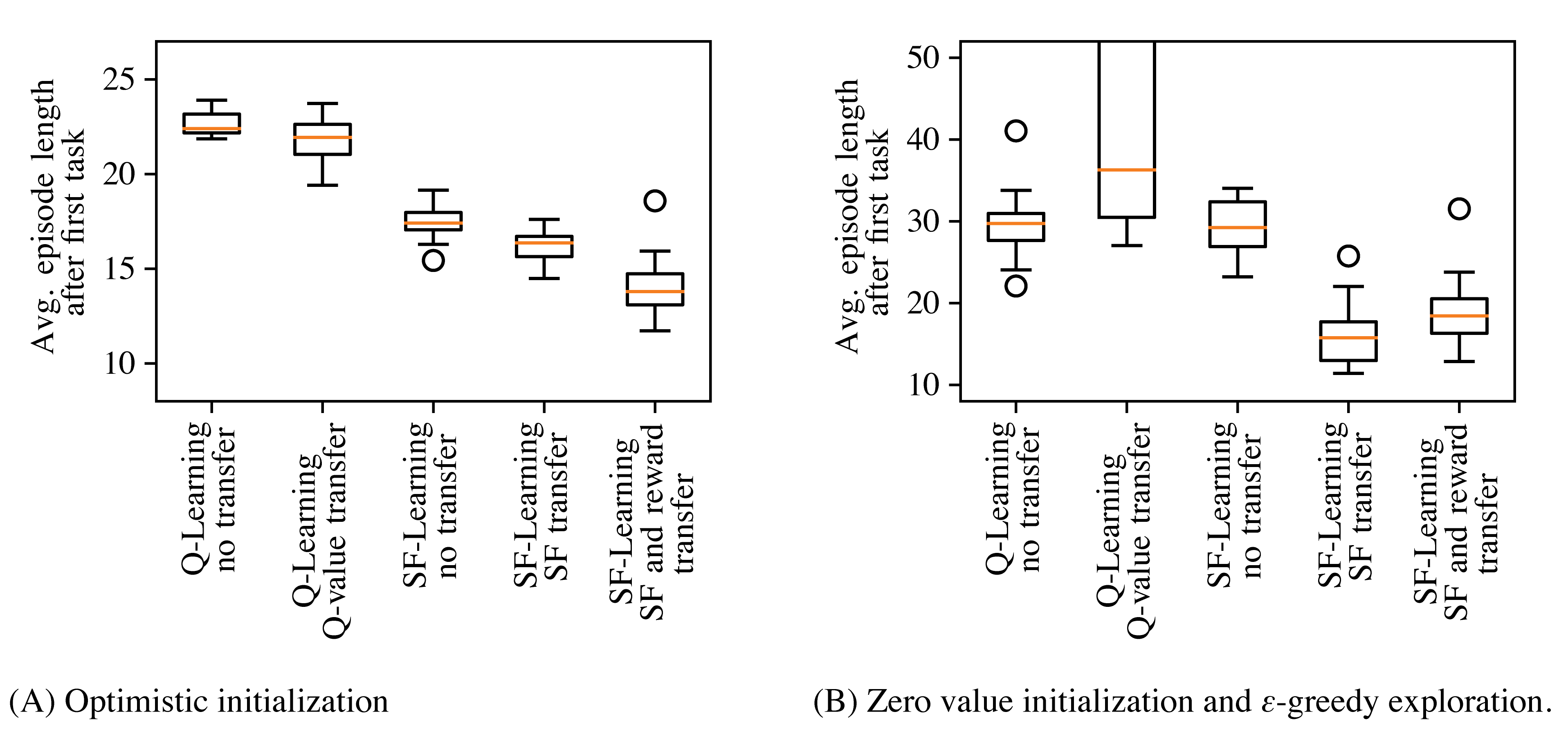

Supplement: S2 Fig — (A) Episode length of Q-learning and SF-learning under different transfer strategies when optimistic initialization is used. The configuration “Q-Learning Q-val. Transfer” re-used previously learned Q-values. The configuration “SF-Learning SF transfer” only re-uses previously learned SFs while the configuration “SF-Learning SF and reward transfer” re-uses both SFs and the learned one-step reward predictions. (B) In this experiment both algorithms are initialized to produce zero Q-values and an ε-greedy exploration policy is used. This exploration strategy selects actions uniformly at random with ε probability and with 1 − ε probability actions are selected greedily with respect to the current Q-value predictions. At the beginning of training ε = 1 (uniform random action selection) and by episode 80 ε was decreased to zero (greedy action selection) using linear interpolation. (TIF) [file pcbi.1008317.s005.tif]

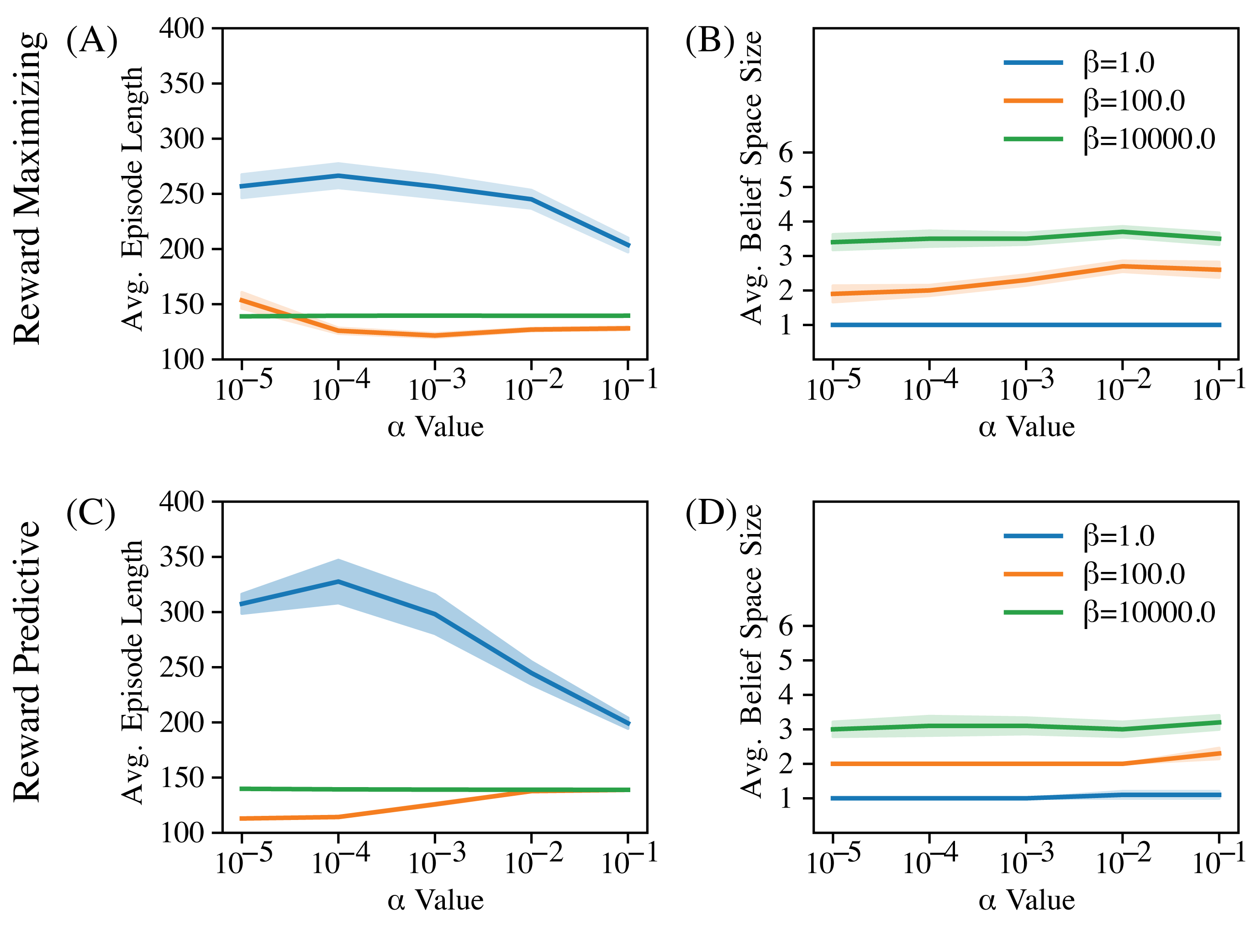

Supplement: S3 Fig — (A) Avg. episode length of the reward-maximizing model. (B) Avg. belief space size of the reward-maximizing model. (C) Avg. episode length of the reward-predictive model. (D) Avg. belief space size of the reward-predictive model. (TIF) [file pcbi.1008317.s006.tif]
