# Supplementary material for: Reward-predictive representations generalize across tasks in reinforcement learning
Source: PLoS Comput Biol. 2020 Oct 15;16(10):e1008317. doi: 10.1371/journal.pcbi.1008317 (PMC7591094; doi:10.1371/journal.pcbi.1008317)
Supplement: Attachment — Submitted filename: review.pdf [file pcbi.1008317.s010.pdf]

Summary
The authors set out to determine what kinds of state abstractions in MDPs support flexible transfer to new environments. They test two rules for generating abstractions: abstractions that support discovery of a policy that maximizes reward, and abstractions that support accurate prediction of a sequence of reward given a random sequence of actions. The agent can learn a set of successor features over these state abstractions which predict the cumulative discounted expected activity of this feature under a policy.

In the first set of results, the authors assess the ability of different abstractions to obtain high reward at transfer. They find that abstractions with a high reward prediction score tend to transfer better to a novel environment. This is the case for both a "column reward" 3x3 grid world and a 3x3 grid world with two random uncorrelated reward locations. In the next results, the authors devise a nonparametric Bayesian inference algorithm for arbitrating over multiple abstractions to conduct transfer with multiple state abstractions based on the loss (maximizing or predictive) associated with each abstraction. They find that using l_predictive allows the agents to identify the "correct" abstractions while l_maximizing leads to the agent requiring a large number of abstract states. Finally the authors describe an architecture that learns to transfer multiple state abstractions. They find that the maximizing abstractions generalize more rapidly to a more diverse set of environments.

The main points the paper has interesting results on a topic of high interest. However, there remain some substantial questions about the work, and room for improvement on clarity and exposition.

Major Comments:
First major comment regards clarity – while the main idea was clearly communicated clear, I had a lot of trouble following exactly how results were generated. There were some points that were either missing from the main text (or possibly just hard to piece together) (e.g. how reward predictions + reward maximizing policies were generated, actions available at boundary states in grid world, train/test splits). Furthermore, I think readability would be significantly enhanced if the paper were more concise. While it is good that the authors build up the model gradually, it seems like the same ideas are repeated but with apparent differences between them that makes it difficult to establish the exact method (as one example, reward predictive is introduced far before it is clarified that it refers to predicting sequences rather than cumulants or immediate reward).

One major point I was unclear on was the exact mechanism of reward prediction + optimal policy generation from SFs and the exact mechanism of generating a new policy in the transferred context. The authors mention that it is zero shot generalization, and it is important to see how the policy is composed from these abstractions.

The second was that I had trouble understanding how to think about the comparability between reward maximizing and predicting representations since they seem different in a number of ways. First, it seems that reward predicting representations should capture strictly more information than reward maximizing. This isn't strictly a benefit, since it might capture more unnecessary information which might make transfer slower, but it makes them hard to compare. Second, it was not clear if there is significance to the fact that reward is predicted rather than some other environmental feature. Third, if I understand correctly, the abstractions are essentially being evaluated on their ability to capture information about different policies: the optimal reward maximizing policy in one case, and a random walk in

the other. Since random walk policies are known to generalize well (since they're a maximum entropy prior), this might be the underlying mechanism of its generalizability. Essentially these abstractions are learned off policy for a good prior default policy – it would be interesting to compare them to random walk SFs.

Minor:
A lot of the paper focuses on evaluating abstractions rather than determining how to form them. This should be clarified as a key objective.

How were train/test (or familiar/transfer) environments delineated?

Use of policy/transition was sometimes unclear – hard to know if it was referred to the transition structure of the MDP or the transitions of the optimal policy specifically.

Related work on universal successor feature approximators (Borsa et al 2018) likely relevant.

don't understand how reward prediction can be better than state prediction, since state prediction is reward prediction in the hypothetical where each state is rewarding.

main finding should be about settings/assumptions under which reward predictive -- rather than state predictive -- generalize better?

In grid world, are rewards absorbing? What happens at boundary states (eg if you go right from rightmost column?)
