# Supplementary material for: Reward-predictive representations generalize across tasks in reinforcement learning
Source: PLoS Comput Biol. 2020 Oct 15;16(10):e1008317. doi: 10.1371/journal.pcbi.1008317 (PMC7591094; doi:10.1371/journal.pcbi.1008317)
Supplement: Attachment — Submitted filename: response.pdf [file pcbi.1008317.s011.pdf]

Lucas Lehnert
PhD Candidate
Computer Science Department
Brown University
Providence, RI 02912-1910
USA

July 15, 2020

Dear Dr. Gershman,

Thank you very much for considering our manuscript, entitled "**Reward-predictive representations generalize across tasks in reinforcement learning**" for publication in *PLOS Computational Biology*.

We would like to thank you and the three reviewers for the thoughtful comments and the opportunity to strengthen and expand our manuscript. Below, we provide a point by point response to each comment. We believe that we have addressed each point adequately and the manuscript has benefited from this process. All changes are all highlighted in blue in the manuscript file `manuscript_r1_diff.pdf`.

We hope that you and the reviewers will find our revisions acceptable.

Sincerely,

Lucas Lehnert, Michael Littman and Michael Frank

Thank you for the thoughtful feedback and comments. As requested, we now provide all program code and the generated simulation data publicly available on github at this address: `https://github.com/lucaslehnert/rewardpredictive.git` To ensure reproducibility of the figures included in the manuscript, we have revised our code and set random number seeds in all simulation scripts. While this leads to minor quantitative changes in Figs 3, 5, 7A, and S6, these changes are not qualitative and do not impact the resulting analysis.

All line numbers mentioned below refer to the manuscript file `manuscript_r1_diff.pdf`.

1 Comment from Editor

> The comments are lucid and I won't reiterate them here. However, I'd like to amplify a point made by one of the reviewers: the manuscript as currently written has very little connection to biology. This would not be a problem if you were submitting to a computer science journal, but this is a biology journal. In order for your manuscript to be publishable in PLOS CB, you need to engage with the relevant biology more directly. In particular, how might your proposed model be implemented in the brain? Is there any evidence for this implementation? As I'm sure you know, there has been a concerted effort to find behavioral and neural correlates of the successor representation; would your model make the same predictions or different ones? I presume different, but it's hard for me to tell exactly what the model would say about those experiments. All that being said, you don't need to dramatically change the length or structure of the paper; it would be sufficient to address these issues in the Discussion. I just want to emphasize that however you address them, you should speak directly to empirical facts about biology and not just general concepts.

Thank you for raising this point. Existing experiments searching for neural and behavioral correlates of the SR [1, 2] have not varied both rewards and transitions, because (unlike the reward-predictive model), the SR is not robust to these changes across environments. Our work motivates the development of targeted experimental designs that would test if human subjects can reuse a latent structure that is present in a set of tasks despite variations in transitions and rewards. For example, one could design a human subject study similar to [3] where participants solve a sequence of grid-world navigation problems, but augment the design to test if subjects reuse a latent structure present in a set of tasks despite variations in transitions and rewards, similar to the task sequence presented in Fig 5. As illustrated in Fig 6, the specific pattern of generalization across tasks is predicted to vary depending on whether agents use reward-predictive state abstractions or re-use SR abstractions. Thus, our work provides a concrete testable behavioral prediction that would discriminate between our work and existing work.

As to the neural correlates, we are beginning to explore potential implementations, and our

work here already highlights some relevant predictions, which we now further elaborate in the manuscript. For example, offline hippocampal replay has been proposed to reflect sampling from a model to train model-free RL and facilitate planning [4–7]. Our work provides a predicted amendment to this notion: we suggest that replay may be prioritized in such a way that facilitates the construction of reward-predictive state abstractions. In our work on learning (Sec 3.3), while the agent is first interacting in a novel MDP, it retains an identity (i.e., un-compressed) state abstraction. Only after sufficiently learning and interacting in this task, the agent can then construct a new state abstraction that can be used for planning in the future. Indeed, for efficient learning and generalization, retaining the identity map while learning is critical; otherwise the agent is likely to create a sub-optimal abstraction that will not generalize. We suggest that the online use of the identity matrix may depend on the highly pattern-separated and conjunctive representations in the hippocampus, whereas the more abstract representations that facilitate generalization and transfer may be cortical [8]. Moreover, we speculate that one way this abstraction could be learned offline would be if, during replay, hippocampal events could be sequentially sampled from regions of the state space that are most similar in a reward-predictive sense (i.e., those that incur the least reward-predictive loss). In this way, an abstract graph-like structure suitable for future planning could be constructed [9,10] but further augmented so that it does not depend on temporal adjacency of transitions within the graph itself, but rather in terms of the ability to predict future expected reward sequences – facilitating a deeper form of transfer. This reward-predictive loss function for guiding replay may also shed light on recent studies in rodents demonstrating that replay is biased toward recently received rewards (e.g., food) rather than those that are currently desired (e.g., water) after revaluation, even though behavior is directed toward the desired one [11]. While this pattern is counter-intuitive from the perspective that replay is used for future planning, it accords with that expected from an algorithm that compresses the state space based on reward-predictive representations, where reward is defined by the previously experienced reward function.

The revised manuscript discusses these connections to biology in lines 840ff.

2 Comments from Reviewer 1

```
Reviewer #1:  Lehnert and colleagues discuss state abstractions and
their role in future performance on RL tasks.  Specifically, define
separate state abstractions into those that support reward maximization
and those that support reward predictions in current tasks.  They
demonstrate that reward predictive state abstractions are more beneficial
to reward maximization on future tasks than reward maximizing state
abstractions are.  More prosaically, they suggest a good state abstractions
are those that preserves the causal chain of rewards.
They first demonstrate this point by enumerating all possible state
abstractions in 9-state MDPs and showing that the state abstractions
that are empirically reward predictive transfer better than the reward
```

maximizing ones.
The authors subsequently develop a Bayesian model to infer upon state abstractions for appropriate reuse between tasks. They show that the Bayesian model equipped with the reward predictivity (from the linear successor feature model (LSFM) arXiv preprint of Lehnert & Littman 2019) of each state abstraction is able to successfully infer that only two state abstractions are needed, whereas a Bayesian model equipped reward maximizing information is not.
Lastly, the authors consider learned state abstractions. Reward maximizing state abstractions are obtained from clustering Q-values. Reward predictive abstractions are learned via gradient descent on a LSFM objective function, and subsequent clustering of states. They demonstrate that, along with the above Bayesian model, reward predictive state abstractions allow for faster learning of new RL tasks that share the same underlying state space.
COMMENTS
Overall, I like this paper and think it should be published. Nevertheless, there are a few places where the impact, broader appeal and clarity of this paper could be improved upon:
1. I had some difficulty understanding the results of the second experiment. For each of the two state abstractions (\phi_A and \phi_B) that define the task, there are many state abstractions that are just as reward predictive as each other. For example, the identity abstraction is always as reward predictive as \phi_A or \phi_B. With this in mind, I don't understand how the reward predictive Bayesian model only uses two abstractions even when \alpha is high. I can think of two possible explanations, neither of which I could find in the paper. 1) You only consider state abstractions that compresses the 9-state MDP to a 3-state MDP. 2) The loss l_{predictive} contains more information than reward predictivity i.e. it promotes state space compression. Some clarity on these results would be helpful.

Thank you for bringing this to our attention. The first case applies to the results presented in Sec 3.2 (second experiment): The model considers only state abstractions that compress a 9-state MDP into a 3-state MDP. We revised the manuscript to explain this in lines 443ff.

2. Following on from the second point above, the notion of reward predictive state abstractions seemed to be highly tied to LSFMs. Is it really the reward predictivity that's driving things or LSFM? For example, if Figure 5 was repeated but using the empirical definitions of reward maximization and reward predictivity from Fig 3 would you get the same results? If LSFM is critical, then this should be mentioned and a more thorough introduction to them may be necessary.

Thank you for raising this point. LSFMs use a loss objective that upper-bounds the reward-sequence prediction errors of a state abstraction. Generally speaking, the LSFM loss objective may not identify the state abstraction that has the lowest possible reward-sequence prediction error out of all possible state abstractions. There is one exception to this rule: If the MDP can be compressed using a state abstraction that has zero reward-sequence prediction errors, then the LSFM loss objective will be zero for every state abstraction that has zero reward-sequence prediction errors. In this case, the LSFM loss objective can be used to identify every state abstraction that has zero reward-sequence prediction loss. This property is proven by the LSFM Bisimulation Theorem [12, Theorem 2].

The simulation in Fig 5 and any other simulation that uses LSFMs falls into this case, because, for each MDP, there exists at least one state abstraction with zero reward-sequence prediction errors. If the simulation in Fig 5 was repeated using the empirical definition of reward-maximizing or reward-predictive properties, then one would obtain the same results.

The submitted manuscript expands this point in lines 480ff.

> `3. The discussion on biological relevance is fairly limited and the authors do not go so far to suggest possible experiments. A more thorough discussion with behavioral and neural experiments would be a nice addition. Perhaps there are behavioral errors that distinguish reward predictive state abstraction from reward maximizing ones?`

Thank you for raising this point. As stated in the response to the editor, existing experiments searching for neural and behavioral correlates of the SR [1, 2] have not varied both rewards and transitions, because (unlike the reward-predictive model), the SR is not robust to these changes across environments. Our work motivates the development of targeted experimental designs that would test if human subjects can reuse a latent structure that is present in a set of tasks despite variations in transitions and rewards. For example, one could design a human subject study similar to [3] where participants solve a sequence of grid-world navigation problems, but augment the design to test if subjects reuse a latent structure present in a set of tasks despite variations in transitions and rewards, similar to the task sequence presented in Fig 5. As illustrated in Fig 6, the specific pattern of generalization across tasks is predicted to vary depending on whether agents use reward-predictive state abstractions or re-use SR abstractions. Thus, our work provides a concrete testable behavioral prediction that would discriminate between our work and existing work.

As to the neural correlates, we are beginning to explore potential implementations, and our work here already highlights some relevant predictions, which we now further elaborate in the manuscript. For example, offline hippocampal replay has been proposed to reflect sampling from a model to train model-free RL and facilitate planning [4–7]. Our work provides a predicted amendment to this notion: we suggest that replay may be prioritized in such a way that facilitates the construction of reward-predictive state abstractions. In our work on learning (Sec 3.3), while the agent is first interacting in a novel MDP, it retains an identity (i.e., un-compressed) state abstraction. Only after sufficiently learning and interacting in this task, the agent can then construct a new state abstraction that can be used for planning in

the future. Indeed, for efficient learning and generalization, retaining the identity map while learning is critical; otherwise the agent is likely to create a sub-optimal abstraction that will not generalize. We suggest that the online use of the identity matrix may depend on the highly pattern-separated and conjunctive representations in the hippocampus, whereas the more abstract representations that facilitate generalization and transfer may be cortical [8]. Moreover, we speculate that one way this abstraction could be learned offline would be if, during replay, hippocampal events could be sequentially sampled from regions of the state space that are most similar in a reward-predictive sense (i.e., those that incur the least reward-predictive loss). In this way, an abstract graph-like structure suitable for future planning could be constructed [9,10] but further augmented so that it does not depend on temporal adjacency of transitions within the graph itself, but rather in terms of the ability to predict future expected reward sequences – facilitating a deeper form of transfer. This reward-predictive loss function for guiding replay may also shed light on recent studies in rodents demonstrating that replay is biased toward recently received rewards (e.g., food) rather than those that are currently desired (e.g., water) after revaluation, even though behavior is directed toward the desired one [11]. While this pattern is counter-intuitive from the perspective that replay is used for future planning, it accords with that expected from an algorithm that compresses the state space based on reward-predictive representations, where reward is defined by the previously experienced reward function.

This revised manuscript incorporates this discussion in lines 840ff.

```
4. This wasn't the clearest / easier-to-read paper. If for each
figure the sub figures had labels A, B, C etc it would make all figures
much easier to parse and refer to in the text. There are also lots
of repetitions in the text e.g. Line194-197 repeats what was said
2 sentences before.
```

Thank you for pointing us to this issue. We have updated Figs 1, 2, 3, and 4, which now contain sub-figure labels and captions. We have also removed the sentence in lines 196ff. to avoid repetitions.

```
MINOR COMMENTS
Figure 7c is mislabeled
```

Thank you, this typo is fixed now.

```
Tau/beta in Figure 5
```

Thank you, this typo is fixed now and tau is replaced with beta.

```
Line 589 { state 0 and state 100?
```

Thank you, this typo is fixed now.

```
Finally, I don't want to make undue additional work for you guys,
so feel free to say the above suggestions don't makes sense etc.
```

3 Comments from Reviewer 2

```
Reviewer #2: I found the topic of this paper really interesting
and exciting. I think that, in general, a formal account of what
makes useful state abstractions of the type presented in this paper,
will be very important for the psychology and neuroscience of human
abilities to generalize experience from one task and apply it to
new tasks. The objective that such representations should be able
to predict arbitrary reward sequences, and that the linear successor
feature models can be used to identify representations that fulfill
this objective, is an interesting insight that I think will be thought
provoking for future work in this area.

Major comments:

My only major comment for the paper regards the claim that the approach
of transferring reward predictive state representations is well suited
for situations in which the task transition structure changes between
tasks. The paper makes a point here to contrast this ability with
that of the successor representation/features approach which is unsuited
for this situation. I'm not sure I was convinced however of the
clear dominance of this approach however by any computational experiments
that they presented. In particular, the authors only test this ability
in families of MDPs that have a common underlying generative process,
in which the difference they share a "true" state abstraction (e.g.
Fig 3 b). The only experiment in which there is no true underlying
abstractions is presented in (Fig 3c). However, for this experiment,
only the reward function changes, but not the transition function.
So, I think it might be useful to provide a simulation similar to
figure 3c, but for a situation where transitions change between tasks,
but there is no singular underlying generative abstraction to see
how the reward predictive abstraction approach performs.
```

Thank you for pointing out this gap in the presentation. We have conducted a modified version of the simulation in Fig 3C that now considers a transfer experiment where both transitions and rewards change but there is also no underlying generative state abstraction present. The transition function change was incorporated by placing barriers (black lines in Fig 3(c)) at different locations in the grid. While the significance levels change, the revised figure presents a similar result despite the changes in transitions. The submitted manuscript includes a revised version of Fig 3.

```
Additionally when successor features approach and the reward predictive
state representation approach are compared in Fig 6, it is not clear
that reward predictive representations perform better than the successor
features approach, even though lots of transitions change in this
```

task. So, it might also be useful to present some sort of task where the reward predictive state representation approach is able to perform much better than SF due to its ability to transfer state abstractions between tasks with transition changes.

Thank you for bringing this to our attention. In the grid-world tasks, we found that our SF-learning algorithm implementation in combination with the initialization heuristics converges faster to an optimal policy than the Q-learning algorithm. (Note that all hyper-parameters were optimized using grid search, as reported in S3.) In Fig 7A, the reward-predictive model transfers state abstractions across tasks but still uses the slower Q-learning algorithm on the constructed latent state space.

To obtain a fairer comparison between transferring SFs and the reward-predictive model, the revised manuscript replaces Fig 7B and includes a new simulation that combines the reward-predictive model with the SF-learning algorithm. Note that the reward-predictive model in Fig 7B only transfers reward-predictive state abstractions between tasks and does not transfer any previously learned successor features themselves. The blue curve in Fig 7B plots the average episode length when the SF-learning algorithm is used to find an optimal policy. In this simulation, the SF-learning algorithm does not transfer a representation and instead resets its weights when switching between tasks. The orange curve plots the average episode length when the SF-learning re-uses previously learned SFs instead of resetting its representation. Fig 7B demonstrates that re-using SFs degrades performance on the maze task sequence while the reward-predictive model outperforms a SF-learning baseline.

As proposed in your comment, we are including a new example to more clearly illustrate the differences between the reward-predictive model and transferring SFs (please refer to Sec 3.4 of the revised manuscript). In this example, we consider the task of playing a scale on a guitar. To play the note "C" on a guitar, for example, one has to hold down a finger in one out of multiple possible locations on the fret board. Fig 8A of the revised manuscript illustrates how we encode the fret-board position to play a note as the task state. In this task, a reward-predictive state abstraction models how different fret-board positions (different states) correspond to playing the same note by mapping these board positions to the same latent state. On a sequence of two scale-playing MDPs, we demonstrate that the reward-predictive model outperforms an algorithm that transfers SFs on the second task. The performance discrepancy in Fig 8B comes about because SFs and reward-predictive state abstractions model different aspects of an MDP.

Minor Comments: Then, I have a number of minor comments mostly regarding clarity:
Section 2: I think the discussion of LSFMs being used to construct reward predictive state abstractions will be confusing to lots of readers. I found myself needing to double back and forth with the supplement section, then also read parts of the prior paper (Lehnert and Littman, 2019) to feel like I understood it. In particular, I think it's not clear from the main text what precise role successor features play in the construction/learning of state abstractions/partitions.

```
I'd recommend adding a bit here more fully spelling out more precisely
how successor features are used to construct latent state partitions,
and also providing more intuition for the central theorem in Lehnert
and Littman, 2019, which relates error over reward predictions to
errors in successor features.  Relatedly here, in terms of understanding
the use of the SF for defining reward-predictive state partitions,
I felt like I didn't understand why it was better to be able to predict
the full successor representation from each latent state (feature)
as opposed to just being able to predict just one-step transitions.
```

Thank you for bringing this to our attention. In fact, [12] shows that one could also learn a model that predicts only the expected next state feature vector. (The expectation of the vector that is reached after selecting an action at a state.) This model is called a Linear Action Model (LAM). While both models can be used to learn reward-predictive state abstractions, LSFMs and LAMs are not equivalent and we have found it easier to use LSFMs in practice. The revised manuscript expands the discussion of LSFMs and discusses this connection in lines 253ff as well as supporting text S2.

```
Section 3.2:  Fig 4:  In the triangle MDP plot, I think the left
action from state 1 to 2 is mislabeled (a instead of c).
```

Thank you, the typo in Fig 4 is fixed now.

```
Fig 5:  in the legend to the bar plot, should this be beta (instead
of tau)?
```

Thank you, the typo in Fig 5 is fixed now.

```
Fig 5:  For this whole figure, I think I understand this, but also
found it somewhat confusing.  Just for clarity, the text suggests
that the main point of this figure is that when the CRP prior is
completely ignored, the reward predictive agent gets the correct
state abstraction, whereas the reward-maximizing agent does not.
For additional parts of the figure however (which vary parameter,
etc.), it looks like the approaches do not produce super clear differences
in their results.  Is this correct?
```

Thank you for raising this point. Yes, this is correct. The description of this simulation was expanded in lines 450ff.

```
Fig 5:  Is the identity state abstraction excluded from this analysis?
Otherwise, why is it not the top scoring abstraction for both measures?
Does anything about the scoring process favor state abstractions
with fewer partitions?
```

Thank you for bringing this to our attention. The first case applies to the results presented in Sec 3.2 (second experiment): The model considers only state abstractions that compress a 9-state MDP into a 3-state MDP. We revised the manuscript to explain this in lines 443ff.

```
Section 3.3 Did I understand this correctly that updates to Pr(psi|Mt,Bt,Ct)
only take place offline, between tasks?  I think it would be useful
to present exactly at what time point each model structure (beliefs
about latent states, Q values, etcl) are updated from experience.
Apologies if I missed this.
```

Thank you for the opportunity to expand on the offline aspects of the algorithm, which we think is important and relevant for biology. Yes, the model is updated off-line after experiencing 200 trials in a specific task. After collecting this data set, the data set is used to find a state abstraction. Intuitively, one can think of this as a form of replay: First, the agent learns how to behave in a task using model-free learning based on the identity map (e.g., a pattern-separated hippocampal represenation of space). After learning in this task, the agent replays what it has seen before to construct a state abstraction offline. This state abstraction can then be re-used when learning in the next task. The revised manuscript explains this idea in lines 564ff.

```
Supporting information 2:  For equation (1) , (2) and (3), shouldn't
P and M be exponentiated with each summed component?
```

Thank you for pointing this out. We revised supporting text S2 and the presented equations.

```
Lastly, I'd would also advocate, if there is not a word limit here,
that aspects of the supplement that describe the simulation protocols
should be moved to a Methods section (not in a separate document
from the rest of the paper) so that readers will not have to download
new documents to access these sections.
```

Thank you for raising this point. The revised manuscript expands the description of the simulation protocols in several places, for example lines 564ff., lines 504ff., lines 443ff., and lines 480ff.

4 Comments from Reviewer 3

```
Summary
The authors set out to determine what kinds of state abstractions
in MDPs support flexible transfer to new environments.  They test
two rules for generating abstractions:  abstractions that support
discovery of a policy that maximizes reward, and abstractions that
support accurate prediction of a sequence of reward given a random
sequence of actions.  The agent can learn a set of successor features
over these state abstractions which predict the cumulative discounted
expected activity of this feature under a policy.

In the first set of results, the authors assess the ability of different
abstractions to obtain high reward at transfer.  They find that abstractions
with a high reward prediction score tend to transfer better to a
```

```
novel environment.  This is the case for both a ``column reward''
3x3 grid world and a 3x3 grid world with two random uncorrelated
reward locations.  In the next results, the authors devise a nonparametric
Bayesian inference algorithm for arbitrating over multiple abstractions
to conduct transfer with multiple state abstractions based on the
loss (maximizing or predictive) associated with each abstraction.
They find that using l_predictive allows the agents to identify the
``correct'' abstractions while l_maximizing leads to the agent requiring
a large number of abstract states.  Finally the authors describe
an architecture that learns to transfer multiple state abstractions.
They find that the maximizing abstractions generalize more rapidly
to a more diverse set of environments.

The main points the paper has interesting results on a topic of high
interest.  However, there remain some substantial questions about
the work, and room for improvement on clarity and exposition.

Major Comments:  First major comment regards clarity - while the
main idea was clearly communicated clear, I had a lot of trouble
following exactly how results were generated.  There were some points
that were either missing from the main text (or possibly just hard
to piece together) (e.g.  how reward predictions + reward maximizing
policies were generated, actions available at boundary states in
grid world, train/test splits).  Furthermore, I think readability
would be significantly enhanced if the paper were more concise.  While
it is good that the authors build up the model gradually, it seems
like the same ideas are repeated but with apparent differences between
them that makes it difficult to establish the exact method (as one
example, reward predictive is introduced far before it is clarified
that it refers to predicting sequences rather than cumulants or immediate
reward).
```

Thank you for bringing these points to our attention.

We revised the definition of reward-predictive state abstractions in line 68 and more clearly state that reward-predictive state abstractions allow an agent to predict future reward sequences.

The presented simulations generate state abstractions in one of two ways:

1. Enumerate all possible state abstractions. This method is used for all simulations in Sec 3.1 and 3.2.
2. Learn a state-abstraction function from transition data. This method is used in Sec 3.3.

Reward-predictive state abstractions can be learned using LSFMs. Reward-maximizing state

abstractions can be computed by clustering states with approximately the same Q-values into one latent state. The resulting state abstraction, called an approximate Q^* similarity abstraction, allows an agent to find an optimal policy in one task [13]. To clarify which method is used when, we revised the writing in lines 211ff., 564ff., 330, and 441.

Regarding how policies were generated: In Secs. 3.1 and 3.2, a policy was computed by first computing the latent state-space transition and reward tables, which is referred to as the *abstract MDP* in the state-abstraction literature [14]. (Please refer to supporting text S1 for a detailed description of how these tables are computed.) This abstract MDP is then given as input to the value-iteration algorithm to compute a policy that is optimal with respect to the abstract MDP. Depending on how the latent state space is constructed, this policy may or may not be optimal in the original MDP. Please also refer to lines 299ff and lines 450ff where this approach is outlined as part of the simulation protocol. We also expanded this argument in lines 451ff. In Sec 3.3, the reward-predictive and reward-maximizing models learn an optimal policy by simulating the Q-learning algorithm on the latent state space. Specifically, if Q-learning would normally observe a transition (s, a, r, s'), the algorithm now observes a transition $(\phi(s), a, r, \phi(s'))$. Because Q-learning caches Q-values for latent states (and multiple states map to the same latent state), the agent now generalizes Q-values across multiple states and can thus converge faster. Simulating online learning algorithms in this way is common practice in the state-abstraction literature [15]. This argument is described in lines 575ff. and we have also expanded the description in lines 583ff.

Please refer to our comments below on boundary conditions and train/test set splits.

We hope that these revisions address the mentioned concerns.

> `One major point I was unclear on was the exact mechanism of reward prediction + optimal policy generation from SFs and the exact mechanism of generating a new policy in the transferred context. The authors mention that it is zero shot generalization, and it is important to see how the policy is composed from these abstractions.`

Thank you for raising this point. Please also refer to our response to the previous paragraph. A reward-predictive state abstraction only models equivalences between states, but not the mapping of latent states to actions or Q-values. While a reward-predictive state abstraction affords zero-shot generalization, the mapping from latent states to optimal actions or Q-values does not afford zero-shot generalization and has to be re-learned from scratch. This idea is described in lines 734ff. A (reward-predictive) state abstraction ϕ can then be used to learn an optimal policy by first mapping each observed state s to its latent state $\phi(s)$ and feeding this latent state $\phi(s)$ as input into the Q-learning algorithm. This idea is described in lines and 530ff. As mentioned above, we also expanded the description of these aspects.

> `The second was that I had trouble understanding how to think about the comparability between reward maximizing and predicting representations since they seem different in a number of ways. First, it seems that reward predicting representations should capture strictly more information than reward maximizing. This isn't strictly a benefit, since it might capture more unnecessary information which might make transfer`

```
slower, but it makes them hard to compare.  Second, it was not clear
if there is significance to the fact that reward is predicted rather
than some other environmental feature.
```

Thank you for the opportunity to expand on the differences between reward-maximizing and reward-predictive state abstractions. Yes, the latent state space constructed by a reward-predictive state abstraction captures more detail about an MDP than a reward-maximizing state abstraction would, and this can be observed in Fig 2. (Please also refer to lines 267ff at the end of the Fig 2 caption.) While a reward-maximizing state abstraction can accelerate learning significantly, such a state abstraction can only be constructed if the optimal policy is already known for this specific task. Because one would need to know the optimal policy or value function before one could construct a reward-maximizing state abstraction, the utility of reward-maximizing state abstractions in learning that same policy is limited. The main argument of the manuscript is that reward-predictive state abstractions capture aspects of an MDP that generalizes across different tasks. Critically, a previously learned reward-predictive state abstraction can be reused to learn an optimal policy in a previously unseen task. This dichotomy in the learning objective is discussed at the beginning of the discussion Sec 4, lines 799ff.

```
Third, if I understand correctly, the abstractions are essentially
being evaluated on their ability to capture information about different
policies:  the optimal reward maximizing policy in one case, and
a random walk in the other.  Since random walk policies are known
to generalize well (since they're a maximum entropy prior), this
might be the underlying mechanism of its generalizability.  Essentially
these abstractions are learned off policy for a good prior default
policy - it would be interesting to compare them to random walk SFs.
```

While LSFMs extract a reward-predictive state abstraction from a random-walk SF, by the LSFM Bisimulation Theorem [12, Theorem 2] the SFs for any arbitrary abstract policy can be used to identify a reward-predictive state abstraction. In fact, Fig 2(d) plots a random walk SF. An abstract policy is a policy defined on the latent state space, i.e. every policy $\pi_\phi : \mathcal{S}_\phi \to \mathcal{S}$.

More importantly, reward-predictive state abstractions generalize across all possible abstract policies and are not constrained to only predict a random walk. This property is the main difference between reward-predictive state abstractions and SFs, because SFs are tied to a specific policy while reward-predictive state abstractions are not.

Intuitively, reward-predictive state abstraction can be used to predict the value of any arbitrary abstract policy by first predicting which reward sequence a specific policy generates and then computing the discounted sum over this reward sequence. This intuition is formalized in [12, Theorem 4]. The revised manuscript incorporates this point in lines 245ff.

```
Minor:  A lot of the paper focuses on evaluating abstractions rather
than determining how to form them.  This should be clarified as a
key objective.
```

Thank you for bringing this to our attention. While the manuscript's main focus is not on a specific abstraction learning algorithm, the goal is to compare different abstraction types and to determine which of the studied abstraction types should be learned by an agent to facilitate re-use across tasks. While the simulations in Secs 3.1 and 3.2 do evaluate different state abstractions, in Sec 3.3 (and Sec 3.4 of the revised manuscript) an offline learning algorithm is used and the emphasis is on how to form and re-use different state abstractions. We revised the manuscript's writing in lines 143ff. to clarify this point.

`How were train/test (or familiar/transfer) environments delineated?`

Thank you for raising this point. In Sec. 3.1, state abstractions are first evaluated on one randomly chosen single task (training or familiar task) and then evaluated on all other transfer tasks (test task). Training and test task are always different. In Secs. 3.2 and 3.3, the non-parametric Bayesian model is exposed to different MDPs in sequence and is signalled when a switch from one task to another occurs. As described in Fig 1, the MDP sequence used in Sec. 3.2 is randomly generated. In Sec. 3.3, the used MDP sequence repeats a previously seen task once, as described in Fig 1. The revised manuscript expands on the simulation protocol in lines 315ff., 420ff., and 564ff. and include this description.

```
Use of policy/transition was sometimes unclear { hard to know if
it was referred to the transition structure of the MDP or the transitions
of the optimal policy specifically.
```

Thank you for pointing this out. We always refer to the transition structure of the MDP itself, not the transition structure under a specific policy. The revised manuscript further clarifies this point in line 109.

```
Related work on universal successor feature approximators (Borsa
et al 2018) likely relevant.
```

Thank you for pointing us to this paper. The universal successor feature approximator approach extends the usual SF framework by re-parametrizing the SFs and defining them as a function $\boldsymbol{\psi}^{\pi}(s, a; \boldsymbol{w})$, where the weight vector $\boldsymbol{w}$ describes a particular MDP. While this approach only requires learning one SF representation function for a family of policies, this model also assumes fixed transition functions. In contrast, reward-predictive state abstractions are robust to variations in transitions and generalize across all possible policies [12].

The revised manuscript includes this argument and a citation [16] in lines 907ff.

```
don't understand how reward prediction can be better than state prediction,
since state prediction is reward prediction in the hypothetical where
each state is rewarding.

main finding should be about settings/assumptions under which reward
predictive -- rather than state predictive -- generalize better?
```

Thank you for bringing this to our attention. In fact, we showed [12] that one could also learn a model that predicts only the expected next state feature vector. (The expectation

of the vector that is reached after selecting an action at a state.) This model is called a Linear Action Model (LAM). While both models can be used to learn reward-predictive state abstractions, LSFMs and LAMs are not equivalents of one another and we have found it easier to use LSFMs in practice. The revised manuscript expands the discussion of LSFMs and discusses this connection in lines 253ff.

> `In grid world, are rewards absorbing? What happens at boundary states (eg if you go right from rightmost column?)`

Thank you for bringing this to our attention. In Fig 1, rewards are absorbing states and a trial is ended once such an absorbing state is encountered. The revised manuscript clarifies this point in lines 615 and 626. On boundary states, the agent does not transition do a different state. For example, if the agent is in state (0, 0) and attempts to transition off the grid, then the next state is (0, 0). The revised manuscript clarifies this point in lines 626ff., 628ff., and 347ff.

References

[1] Momennejad I, Russek EM, Cheong JH, Botvinick MM, Daw N, Gershman SJ. The successor representation in human reinforcement learning. Nature Human Behaviour. 2017;1(9):680.

[2] Russek EM, Momennejad I, Botvinick MM, Gershman SJ, Daw ND. Predictive representations can link model-based reinforcement learning to model-free mechanisms. PLoS computational biology. 2017;13(9):e1005768.

[3] Franklin NT, Frank MJ. Generalizing to generalize: when (and when not) to be compositional in task structure learning. bioRxiv. 2019;doi:10.1101/547406.

[4] Mattar MG, Daw ND. Prioritized memory access explains planning and hippocampal replay. Nature neuroscience. 2018;21(11):1609–1617.

[5] Liu Y, Dolan RJ, Kurth-Nelson Z, Behrens TE. Human replay spontaneously reorganizes experience. Cell. 2019;178(3):640–652.

[6] Schuck NW, Niv Y. Sequential replay of nonspatial task states in the human hippocampus. Science. 2019;364(6447):eaaw5181.

[7] Ambrose RE, Pfeiffer BE, Foster DJ. Reverse replay of hippocampal place cells is uniquely modulated by changing reward. Neuron. 2016;91(5):1124–1136.

[8] Atallah HE, Frank MJ, O'Reilly RC. Hippocampus, cortex, and basal ganglia: Insights from computational models of complementary learning systems. Neurobiology of learning and memory. 2004;82(3):253–267.

[9] Schapiro AC, Turk-Browne NB, Norman KA, Botvinick MM. Statistical learning of temporal community structure in the hippocampus. Hippocampus. 2016;26(1):3–8.

[10] Garvert MM, Dolan RJ, Behrens TE. A map of abstract relational knowledge in the human hippocampal–entorhinal cortex. Elife. 2017;6:e17086.

[11] Carey AA, Tanaka Y, van der Meer MAA. Reward revaluation biases hippocampal replay content away from the preferred outcome. Nature Neuroscience. 2019;22(9):1450–1459. doi:10.1038/s41593-019-0464-6.

[12] Lehnert L, Littman ML. Successor Features Combine Elements of Model-Free and Model-based Reinforcement Learning. arXiv preprint arXiv:190111437v2. 2019;.

[13] Abel D, Hershkowitz DE, Littman ML. Near optimal behavior via approximate state abstraction. arXiv preprint arXiv:170104113. 2017;.

[14] Li L, Walsh TJ, Littman ML. Towards a Unified Theory of State Abstraction for MDPs. In: ISAIM; 2006.

[15] Abel D, Arumugam D, Lehnert L, Littman M. State Abstractions for Lifelong Reinforcement Learning. In: Dy J, Krause A, editors. Proceedings of the 35th International Conference on Machine Learning. vol. 80 of Proceedings of Machine Learning Research. Stockholmsmässan, Stockholm Sweden: PMLR; 2018. p. 10–19. Available from: `http://proceedings.mlr.press/v80/abel18a.html`.

[16] Borsa D, Barreto A, Quan J, Mankowitz D, Munos R, van Hasselt H, et al. Universal successor features approximators. arXiv preprint arXiv:181207626. 2018;.
